# Care and the self: biotechnology, reproduction, and the good life

**DOI:** 10.1186/1747-5341-2-6

**Published:** 2007-05-04

**Authors:** Stuart J Murray

**Affiliations:** 1 Department of English, Ryerson University, 350 Victoria Street, Toronto, Ontario, M5B 2K3, Canada

## Abstract

This paper explores a novel philosophy of ethical care in the face of burgeoning biomedical technologies. I respond to a serious challenge facing traditional bioethics with its roots in analytic philosophy. The hallmarks of these traditional approaches are reason and autonomy, founded on a belief in the liberal humanist subject. In recent years, however, there have been mounting challenges to this view of human subjectivity, emerging from poststructuralist critiques, such as Michel Foucault's, but increasingly also as a result of advances in biotechnology itself. In the face of these developments, I argue that the theoretical relevance and practical application of mainstream bioethics is increasingly under strain. Traditionalists will undoubtedly resist. Together, professional philosopher-bioethicists, public health policymakers, and the global commercial healthcare industry tend to respond conservatively by shoring up the liberal humanist subject as the foundation for medical ethics and consumer decision-making, appealing to the familiar tropes of reason, autonomy, and freedom.

I argue for a different approach to bioethics, and work towards a new way to conceive of ethical relations in healthcare – one that does not presume a sovereign subject as the basis of dignity, personhood or democracy. Instead, I am critical of the narrow instantiations of reason, autonomy, and freedom, which, more recently, have been co-opted by a troubling neo-liberal politics of the self. Thus, I am critical of current trends in medical ethics, often running in tandem with corporate-governmental models of efficiency, accountability, and so-called evidence-based best practices. As an example of such market-driven conceptions of subjectivity, I discuss the paradigm of "self-care." Self-care shores up the traditional view of the self as a free agent. In this sense, self-care is looked upon favourably by mainstream bioethics in its focus on autonomy, while healthcare policy endorses this model for ideological and economic reasons. To contrast this, I propose a different model of care together with a different model of selfhood. Here I develop and apply Foucault's late work on the "care of the self." In this understanding of "care," I suggest that we might work towards an ethical self that is more commensurable both with recent theoretical views on subjectivity and – more pressingly – with the challenges of emergent biotechnologies. I end this paper with a discussion on ethical parenthood, which offers a practical reading of the "care of the self" in relation to new reproductive technologies (NRTs).

## Background: questioning the good life

"The unexamined life is not worth living" [1].

While this essay focuses specifically on our changing sense of self in relation to recent advances in biomedical technologies, in the background looms a larger, more philosophical, question concerning life – *bios*: What lives are worth living, what lives worth preserving and reproducing? Today, in a culture saturated by medical discourses of all kinds, it is difficult to read Plato's remark on the value of the "unexamined life" as anything but menacing. The life worth living is the life that submits to examination – "Like a patient etherised upon a table," as T. S. Eliot has famously written. Michel Foucault captures for us this particularly modern sense of the examination: "The examination combines the techniques of an observing hierarchy and those of a normalizing judgement" [2]. To "examine" one's life today is to submit to medical knowledge and techniques, to evaluations, and to normalizing judgements. It is to be governed by so-called experts, and to be understood in and through recent genomic and molecular vocabularies of biomedicine. Indeed, these terms have come to constitute our norms, and it is by virtue of such terms that we can be said to be a "self" in any meaningful way: these techniques of examination increasingly provide the very modes by which we reflect upon ourselves in the quotidian, the modes by which we are tied to our own recognizable identity, the modes by which we assign meaning and value to life itself.

Medical discourse thus informs one manner in which the self or subject is constituted – and silently comes effectively to constitute itself as a subject. In this sense, medicine operates as a "technology of the self," a nexus of social, political, and historical practices and beliefs that provide the very terms of the self and its self-understanding. In Foucault's words, these "technologies" of the self:

permit individuals to effect by their own means, or with the help of others, a certain number of operations on their own bodies and souls, thoughts, conduct, and way of being, so as to transform themselves in order to attain a certain state of happiness, purity, wisdom, perfection, or immortality. [3]

For Foucault, power bears directly upon the ways in which the self relates to itself, including the kind of identity the self can claim for itself as a "true" identity, and the kind of self this self will strive to be in relation to itself and to others. The self is never at peace, as it were; to be alive means in some sense that the self is always cheating its mortality, transforming itself, developing itself, and working to understand itself; the self uncritically takes up the injunction to be happy, pure, wise, perfect, or immortal, as Foucault says. Stated simply, these are discursive modes of *self*-relation, a sort of reflexivity that has become the cornerstone of modern subjectivity. I shall argue that the ways in which the self now relates to itself specifically as a living, genomic organism have begun to alter the way we conceive of the self. There has been a change, I believe, because the terms and techniques of our self-relation are ever more informed by emergent biotechnologies, especially through popularized genetic paradigms and new reproductive technologies (NRTs). What Foucault famously called the "clinical gaze" is fast being supplanted by the "molecular gaze" [4]; biopolitics – a politics concerned with the life of the population – is being supplanted by "molecular politics" [5].

In this essay, I offer a discussion and a critique of current genetic (or genomic) modes of selfhood, drawing in particular on the ways in which the self relates to itself, how our self-knowledge is variously mediated by a rapidly evolving biotechnological discourse. As a result, we must ask: what relations will guide the ethical norms that inform human political life, human dignity, and the common good, especially when these values have their roots in a traditional humanism and liberalism that has come under attack, in part thanks to these technologies themselves? I argue that human identity is fast becoming a matter of genomics, the identity of the self collapsed into its genetic identity. It is increasingly difficult to identify – even obliquely – an unalterable biological nature: biotechnology promises to intervene at the most intimate and elementary level of life itself. But more than discreetly *organ-izing *the body, biotechnology sets up the very vocabulary in and through which all manner of "life" will have social, cultural, and political significance, ultimately determining the kind of experience we can have of ourselves and of others as living beings whose lives have value. Consequently, our socio-organic relationship to ourselves and to others – and especially to our children – is undergoing a profound transformation. More complexly still, biotechnology is a burgeoning field of research and practice, representing a vast industry spawned in part by the Human Genome Project, including the manifold interests of agribusiness, multinational pharmaceutical corporations, reproductive and therapeutic medicine, and even governmental agencies involved in all aspects of regulating human life, from insurance and public health policy to biological warfare and bioterrorism. Together, we might call this the "biotech apparatus," the background or lifeworld within which human relations unfold, are understood, and can be valued.

For the purposes of this essay, I shall limit myself to a brief discussion of emerging biotechnologies that involve genomic medicine and the biotechnological screening and/or manipulation of the human body at the molecular level. First, we might think of the woman who is genetically tested for the "breast cancer genes," BRCA1 or BRCA2. From these tests, geneticists can offer her statistics concerning her risk for developing breast cancer; and, armed with these statistics, medical practitioners can suggest therapies or strategies for "risk reduction" and self-care. Here, the patient is placed in the impossible situation of having to imagine and relate to a future self, a statistical or actuarial self, a self that does not yet and might never exist, in fact. As a second example, we might consider how emerging reproductive medicine relies on a similar rhetoric of "risk reduction" and "risk management," coupled with the strange temporality of the not-yet, the future baby, a spectre that looms larger than life. Of these new reproductive technologies (NRTs), a paradigm case is preimplantation genetic diagnosis (PGD), in which the preembryo or blastocyst is genetically screened for specific diseases. On the basis of these tests, scientists and parents can then choose to implant only those preembryos deemed to be genetically "normal."

The purpose of this paper is to reflect upon such rhetoric in order to question the norms and constraints that govern self-formation in the context of emergent biotechnologies – norms that inform our human biological heritage and the meaning of "the human" in general. Such a critique is increasingly difficult given what might be called the genetic ideology that currently holds sway in Western culture. As a point of interrogation, what will emerge in this paper are two sharply contrasting models of "care." The first I call "self-care," a model that has dominated public health policy in recent years. "Self-care" relies on a model of selfhood that is drawn from the tradition of liberal humanism: the Enlightened, knowing self, the self that is conceived as the source of its own agency, autonomous, free, and guided by conceptual reason. This is the self that medical ethics typically presumes as foundational: rational, autonomous, and freely able to consent. In what follows, I am highly critical of this "self," and I am equally suspicious of the evidence-based movement in medicine and the so-called "best practice guidelines" that have come to define "self-care" in the biomedical management of this self [6-9]. In contradistinction, I shall propose a second model of care that I borrow from Foucault's ethics – "care of the self." I hope to show how the Foucaultian "care of the self" is incommensurate with the care that we find in the "self-care" paradigm. In other words, the model of selfhood that emerges in Foucault's "care of the self" must be sharply distinguished from the more traditional self of "self-care" conceived under the aegis of liberal humanism. In both instances, while *care *is the fundamental manner in which the self relates to itself and to others, again, these two models of care are not equivalent relations – each relation fosters a distinct kind of self. Only the latter can sustain an ethical politics; only the latter can help us once again to question the good life.

I proceed by offering a brief history of the emergence of "self-care," and take pains to illustrate how its particular conceptions of both "selfhood" and "care" have arisen. I argue that emerging biomedical technologies pose a direct threat to the sovereignty of the traditional "self," the self that is bequeathed to us from liberal humanism. Genomic technologies radically challenge our taken-for-granted notion of a rational, autonomous, and free subject. I therefore see the rhetoric of "self-care" as a response to this crisis – an anxious effort to reinstate a rational, autonomous, and liberal subject both in the name of liberal politics (e.g., through public heath policy) and in the name of bioethics (represented by mainstream analytic philosophy). These efforts notwithstanding, if this liberal humanist subject is dead, then public policy wonks and bioethicists are doing no more than offering life-support to a corpse. Rather than endorse a philosophy of resurrection, we must instead work toward a new and revolutionary conception of the subject – one that would be commensurate with the multiple effects and possibilities of genomic technologies. This is not to invent a subject who would wholeheartedly endorse all manner of biotechnology; rather, it is to imagine a subject for whom biotechnologies might be meaningful, a subject who might enter the discourse of its own subjectivation.

Admittedly, this is a radical claim. If the traditional model of the subject is dead – that is, if the familiar tropes of reason, autonomy, and freedom are no longer taken for granted as foundational, then political liberalism and contemporary forms of Western statehood and governance will lose their hegemonic raison d'être. And this, too, is becoming evident in the anxious governmental efforts to conceptualize and contain both the practices and the effects of new biomedical technologies (most spectacularly, stem-cell research and cloning) – discourses and practices that are transforming our conception of self. Thus, we see the emergence of a "state science" and a "state philosophy" on biogenetics. In the words of Slavoj Žižek, this is "a philosophy that would, on the one hand, condone scientific research and technical progress and, on the other, contain its full impact, i.e., prevent it from posing a threat to the existing ethical coordinates" [10]. In the face of these challenges, I turn to a Foucaultian "care of the self," a form of ethical relation which suggests a different model for both "care" and "selfhood." While Foucault does not provide practical or material answers to the political and philosophical challenges posed by biotechnology, I believe the work from the last years of his life opens for us a new and more promising discourse on the self, a discourse that might offer more just and productive ways to map our ethical and political coordinates in the genetic age. This paper concludes by reflecting on the effects of new reproductive technologies and on ethical parenthood in general; I hope that this will provide one concrete instance of how the "care of the self" might begin to take shape.

## The birth of medical morality: from the good life to the good self

"Everyone must discover that he is in a state of need, that he needs to receive medication and assistance" [11].

At this juncture an historical digression will be necessary if we wish to understand the two senses of care that I am proposing. In this section I shall begin with the historical relationship that Foucault draws between medicine and the self. Foucault charts the rise of medical technologies and the emergence of the modern self, a self defined by the modern liberal values of reason and autonomy. In my own terms, this marks the rise of the self of "self-care" – a paradigmatic self that experiences itself as responsible for its own well-being, morally compelled always to act with "due care" toward itself, morally obliged to avail itself of new biotechnological "resources," and, lest he be perceived as biting the hand that feeds him, to see these "resources" as "empowering," if not "liberating." In a later section I shall deal explicitly with the "care of the self" and how this mode of care fosters a very different model of selfhood.

Foucault's late study of medicine is found predominantly in Volumes 2 and 3 of *The History of Sexuality *series, *The Use of Pleasure *[12] and *The Care of the Self *[11], respectively. His analysis deals mainly with medical technologies in the shift from Ancient Greece in the fourth century BCE to the Golden Age of Rome, the first two centuries of our era. More precisely, Foucault charts the historical shift in the human relation *to *medical technologies, in part, as one way to discuss what he sees as an increasing "problematization" of the self in antiquity (and with obvious implications for the individuated self of modernity). For the Greeks, there was no "problem of the self," properly speaking: it would be wrong to speak of a Greek "self" in the sense that we understand this term. Hence, medical practices in Ancient Greece did not constellate around individuated selves who would experience medicine as a "subjective" intervention in one's health or as a "technology of the self," as we do today. Instead, for the Greeks, medicine was one instantiation of the *technē tou biou*, a *technē *or "technology of life" – how to live and live well, how to live the good life (the thrust of the Plato epigraph on the "unexamined life," which fails to resonate in quite the same way today). However, as Foucault demonstrates, in the Roman period that succeeds classical Greece, this is no longer an accurate description of medicine and the medical experience: the relation is increasingly about the *technē *of the self – in brief, a "technology of the self" that is concerned primarily with how *to be *oneself, the *good self *as opposed to the *good life*. Foucault summarizes: "I think that one of the main evolutions in ancient culture has been that this *techne tou biou *became more and more a *techne *of the self" [13]. Or even more succinctly, he writes: "I think that the great changes which occurred between Greek society, Greek ethics, Greek morality, and how the Christians viewed themselves are not in the [moral] code [i.e., the prohibitions], but are in what I call the 'ethics,' which is the relation to oneself" [13].

What I find remarkable about Foucault's historical study is how it reads today. The reader is invited to read a "history of the present" into Foucault's discussion. Our age of obsessive individualism cannot but resonate with the descriptions that Foucault offers of Roman culture, while our experience fails to resonate with the Greek. Whether we are in ancient Rome or in the modern West, the individuals of each epoch are, each in their own way, in thrall to medical technologies as a defining truth of the self, an answer to the problem of the self – a self which silently asks the question of itself and whose very asking is itself a *technē*, a mode by which the self defines itself. And so while for the Greeks, the question was how to live and live well, for Rome – and for us – life is no longer the "ethical substance" or the fundamental question, but selfhood is that substance. Note the way the self operates as a trope in Foucault's discussion of Roman medicine: "medicine was not conceived simply as a technique of intervention, relying, in cases of illness, on remedies and operations. It was also supposed to define, in the form of a corpus of knowledge and rules, a way of living, *a reflective mode of relation to oneself, to one's body, to food, to wakefulness and sleep, to the various activities, and to the environment*" [11] (emphasis mine). This remains a compelling description of medicine and subjectivity today.

What emerges from Foucault's study of medicine in antiquity is that since Roman times medical technologies have been used as a way for the self to work on itself. For the ancients, medicine was a solution to a certain ethical problem of the self, one answer to those social and political questions that an emergent self began to pose. Medicine was one "place," as it were, where that self was elaborated, literally worked-out, when it asked questions about itself and its proper relation to family members and, more broadly, to society. Medicine began to offer a technical means by which the self would relate to itself, prescribing techniques by which that self would be recognized, would experience itself, as the good self. Medicine was therefore a novel aesthetics of existence, one way to break free of past modes of subjectivation, again, by promoting "a way of living, a reflective mode of relation to oneself, to one's body, to food, to wakefulness and sleep, to the various activities, and to the environment" [11]. In Rome, we might say that medicine freed the self. But today, I would argue, we stand in an opposite – and very much more sinister – relation to medical technologies.

Stated in the most polemical terms, modern medicine does not liberate the self – it enslaves it. Today, medicine has become part of the *problem *of the self, and this becomes even more obvious in our genomic era of medicine: who or what am I if I am first and foremost a genetic self; what ethico-political responsibilities do I have to myself, to others, and to my offspring within this paradigm; and what subjective agency is left to me if the sovereignty of the Kantian "I" is displaced from a rational, autonomous self onto a sovereign genetic code that has the first and last word on who I am, what I am, and on who and what I shall become? These are the new problems of the self in a genocentric age. Because genomic vocabularies have so pervaded the public sphere, it is impossible not to understand the self as a problem in these terms:

DNA in popular culture functions, in many respects, as a secular equivalent of the Christian soul. Independent of the body, DNA appears to be immortal. Fundamental to identity, DNA seems to explain individual differences, moral order, and human fate. Incapable of deceiving, DNA seems to be the locus of the true self, therefore relevant to the problems of personal authenticity posed by a culture in which the "fashioned self" is the body manipulated and adorned with the intent to mislead. [14]

If DNA is nothing more than code, a "blueprint" or – better still – a "command" structure for the construction of protein molecules that, in turn, will shape who and what I am, we might well wonder: Who or what commands these commands? Where is the locus of agency? Where is selfhood or subjectivity if, according to the liberal tradition, it is conceived of as agency or autonomy (*auto *+ *nomos*), the capacity to do or to act independently? After all, I cannot be free from my genes. "I" am no longer the source, the foundation of my self. At most, I can *respond *to my genes, after-the-fact. So, if selfhood or subjectivity are traditionally celebrated as the self's capacity to reflect upon itself, this self-reflexivity is now thoroughly undermined by our genes; the richness of subjectivity is displaced by, or, at the very least, dependent upon, the mechanistic world of the gene, a world governed by information, and not by thought.

While we are thoroughly beholden to the terms of modern medicine, and while the self is interpellated as a subject of medical authority, medicine continues to sell itself as "self"-empowering. We are still told that medicine is the cure to the problem of the self, the principal technology by which the self ought to relate to itself, through the body, through our relation with others (infectious diseases), from the minutiae of our sexual lives (STDs, infertility, healthy and happy sex lives, longer and harder erections...), to our "lifestyle" or habits of eating, drinking, smoking, exercise, self-care, and self-discipline. Medicine is now the problem of the self; and medicine, we are told, is the necessary solution to the problem – a problem that this medical discourse has in fact itself secretly produced and systematically obscured (e.g., consider the discourse on "obesity" and its re-packaging as "Metabolic Syndrome"). If medicine is angelic in its promise, it saves us on its own terms ("obesity" and "infertility" are now diseases that medicine and pharmaceuticals promise to "cure"). If anything, today an ethical project worthy of that name would strive to formulate new relations to medicine, new relations to the medical body, new relations to the soul that is constrained to think according to biomedical terminology and to act by perpetuating a medicinal ideology. But the modern self remains constrained by a medical morality: I am morally remiss, my life is a life unworthy of living if I fail to submit to medical examinations, to doctors' and psychiatrists' recommendations, and to proactively minimize my risky behaviours and states-of-mind. I am subject to medico-moral judgement if I fail to exercise "due care," if I neglect my self, if I do not live up to a level of self-care that is sanctioned by medical authorities, government agencies, insurance companies, employers, public health and occupational safety standards, family, friends, and concerned passers-by, who, with a glance, condemn me in my knowledge that this cigarette or cocktail is bad for me and violates life itself. As Novas and Rose comment, the rise of this kind of pressure "reshapes prudence and obligation, in relation to getting married, having children, pursuing a career and organizing one's financial affairs" [15]. "My" health becomes indistinguishable from "public health" or the health of the population: the "individual" is "normalized" and "regularized" by "a technology in which bodies are replaced by general biological processes" [16]. Of course, my "responsibilities" and very shape of these public moral expectations will multiply and shift according to each new biotechnological discovery. It is no longer adequate to follow the Hippocratic principle of "doing no harm"; today I must be proactive, I must do good, and consequently, "for my own good" I must accept on authority what the "good self" would do.

## The ideology of self-care

"Self-care is one of the pillars of health care reform in Canada today. Most self-care is undertaken by people independently of the involvement of health care professionals" [17].

The Government of Canada's public health agency, Health Canada, has defined self-care as follows: "Self-care [is] defined as the decisions and actions taken by someone who is facing a health problem in order to cope with it and improve his or her health" [17]. This description is found in an official policy paper. It is deceptive in its simplicity. It constitutes the individual as *the *locus of decision and action, reinforcing the ideal of the liberal subject so dear to biomedical ethics and liberal politics alike. Self-care presumes a rational, self-reflexive subject who is able to give his or her full – and "informed" – consent. The model relies on the principles of autonomy: either work to raise these individuals to the point where they are self-determining, or else presume them to be self-determining and act in such a way that will prove consistent with this presumption. The problem with the latter, of course, is that these individuals are colonized by discursive models of selfhood and agency that are not, strictly speaking, their own. It is a form of hegemony. "Medical relations of power require this entity, this 'autonomous self,' so that the self-so-constituted can choose to assume a symmetrically lower position in the therapeutic hierarchy for his or her own good" [18].

So while it would appear that the self of self-care is a self that relates to itself freely and transparently, with full knowledge, what proponents of self-care do not say is that this self-self relation is mediated and highly structured, relying on a cadre of so-called experts and technicians, deploying a vocabulary that is sometimes frightening, alienating, and often incomprehensible. There is a "therapeutic hierarchy" that is in some sense inevitable, given the remarkable advances in medical research and the layperson's inability to develop such expertise. And yet this hierarchy is disavowed in favour of a self that is constituted as self-responsible. Responsibility is conceived in economic or entrepreneurial terms [5,15,19]: I, as a patient, am treated foremost as a client who employs expert-providers in my own health care initiatives, to improve my health, to work on my self as if I were not the subject of my own well-being but an object in need of repair or enhancement. Here, the self-self relation is explicitly technologized, instrumentalized. The self relates to itself as through a knowledge economy – I am responsible to "know" my self biomedically, to take decisions and perform "best practice" actions in the project of my own well-being:

Responsibilization operates to individualize social responsibility for managing the risks of biotechnology. Increasingly, individuals are expected, not to discipline themselves, but to manage themselves and the risks that they pose to the wider social good, through accessing and mobilizing the resources and expertise at their disposal in the genetic marketplace. [20]

If I fail to understand what certain risks might mean in real terms, in the terms of my own life, then that failure is somehow mine and mine alone.

But how, we might ask, is a woman expected to understand a genetic test that "predicts" her to have a 28% chance of dying of breast cancer in her lifetime? Suddenly, she is no longer dealing with a "real" medical crisis, but with a potential one. In this gesture, she is quantified, reduced to a bare statistic [21]. Medicine can tell her nothing of the value of her life. She will be at a loss to evaluate the meaning of such personal risk, which she must now assume as her own, in the context of her entire life – a life whose value and duration are themselves impossible factors in the equation. She will be forced to make decisions with unforeseeable consequences, to navigate the unnavigable; every choice will have an existential valence. Even when we oversimplify her decisions, they remain impossible: should she or should she not undergo a prophylactic mastectomy and hysterectomy? Or should she wait and see, and try to live fully under the veil of terror that such a diagnosis will carry with it? Sarah Lochlann Jain refers to this state as "living in prognosis" – "the collision between subjective life and objective death" [22]. In some ways, I suspect that such a life will prove unliveable, now that a positive value has been authoritatively assigned to her risk, based on statistics derived from genetic testing, average life expectancy, typical patient outcomes, and so on. Can we really speak of "informed choice" in this context? Her agency and autonomy are surrendered not just to medical authority, but to a future body-at-risk that is not fully hers, not yet. She is a subject out of time, because "prognostic time constantly anticipates a future" [22] that may or may not include her. Where is her "I," that Kantian locus of subjectivity and reason and truth? And "who" will act, if she can at best *react *to the sovereignty of her elementary particles, to a future that is not yet and may never be in quite this way?

Thus, while there is the ruse (or at least the spectral promise) of epistemic certitude in the rhetoric of self-care – indeed, a promise of biomedical scientificity – self-care fails to offer us anything like the good life and only inaugurates a self that, despite itself, can never be unequivocally good, happy, pure, wise, perfect, or immortal. And yet we strive all the more, wedded to our epistemological worldview, where our own genetic matter is produced "as a field of management and includes practices such as mapping, testing, coding, banking, simulating, and representing" [20]. Despite this surveillance and perpetual self-management, the rhetoric of autonomy and freedom carries the day: it is our ideology, our mantra. We presume the "autonomy of the self" because we cannot imagine an alternative. And such "autonomy" is also the cornerstone of political liberalism and the vast economies we have built. This emphasis on the autonomous individual effectively privatizes and depoliticizes what are properly social and political effects, embodied historical effects whose operational power is summarily masked and disavowed by liberalism.

Challenging such autonomy, ethically or politically, is bound to be met with great resistance. And so we continue to have faith in a self we describe as "free" to choose for itself, an indisputable source of its reason and will. Moreover, we glibly continue to conceive of our technologies as rational extensions of our autonomy, "extensions of man" in body and spirit – from medical technologies to weapons of mass destruction. And to complete this circle, we ardently believe that our technological developments actually increase our choices and, synonymously, our freedom. Freedom, in this model, is naïve: the freedom to choose, the freedom to exercise an "autonomy" that is defined with so much circularity – to have maximum *choice*, and to be free from norms and constraints when we make choices, all the while ignorant of those norms and constraints that inform our desires in the first place. Those who understand freedom in this model tend to see freedom in neo-liberal or "free market" terms: for them, our political freedom is no more than the quantity of choice in our marketplace... fifty brands of shampoo, paper or plastic at the checkout. And so, to conclude this section, I argued above that the ethical basis of traditional liberalism – founded on reason, autonomy, and freedom – has been challenged and outstripped due to burgeoning biotechnologies. In this section, I have argued that neo-liberalism founders for similar reasons; but in a more sinister twist, we are invited to discover an ethics of neo-liberalism in its presumed ethical neutrality – that is, neo-liberalism pretends to be ethically neutral by recasting reason, autonomy, and freedom as parameters of an independently fair and objectively equitable market. This, too, is a ruse, but of greater magnitude [5,9,19].

## "Care of the self"

Socrates: "No physician, in so far as he is a physician, knows himself" (131a) [23].

Here I would like to propose a different model of freedom and subjectivity, derived from Foucault's late work on ethics as "care of the self." Indeed, I hope that this will open us onto a different form of knowledge, a wisdom that is less in thrall to the ruses of autonomy and epistemic certainty – a different mode by which the self will know itself. "Care of the self" is one manner in which the self relates to itself in such a way that the self is not collapsed into the certitude of its genetic identity and what this identity might mean. I prefer to imagine the "care of the self" as a self-self relation that is inventive and open, as a self that questions the norms and constraints in and by which that self is said to be a self in the first place. I see this intervention as a critical move away from a model of "self-care" and "the good self" toward a self that will be in a better position to question the good life. "Care of the self" is incommensurable with "self-care"; indeed, while "care of the self" will not preclude our practises of "self-care," it is a critical attitude that will question the norms and constraints of this movement.

In order to explain the "care of the self" in greater detail, I will need to make a brief digression, to shift rhetorical registers somewhat, to de-familiarize our familiar and fixed ideas on care and medicine. I would like to turn here to a love scene, of sorts, which takes place between Socrates and the young Alcibiades in Plato's dialogue by the same name [23,24]. The dialogue opens with Socrates going to Alcibiades, who, at the age of twenty, is about to enter a career in politics. Alcibiades claims that he is well-prepared to be a wise ruler of the Athenians, and Socrates inquires how he knows this to be true. We might say that Socrates asks Alcibiades by what norms and constraints he will govern wisely, by what principles or rules (*archai*). Importantly, Socrates demonstrates that it is not simply a matter of applying a skill. Indeed, sets of particular skills (*technai*) – whether they are medicine, shipbuilding, or wrestling – will not be sufficient if one is to govern well. Instead, Socrates says, first one must *know oneself*. If one knows oneself, or so the argument goes, then one will understand implicitly, tacitly, that the principles of prudent *self*-governance apply equally well to the just governance of the city-state. In the early pages of the dialogue, Socrates convinces poor Alcibiades, who is initially cocky and prideful, that in fact he is quite ignorant and *un*wise in these matters. Alcibiades's pride soon turns to despair. What follows is a discussion on what it means to know oneself and to govern wisely.

Significantly, the dialogue is *not *primarily concerned with epistemological questions. Epistemology is about what we know, and how it is that we know what we know, and know it with certainty. This knowing self is the self of self-care that I outlined above. Instead, the dialogue is concerned with the Delphic inscription – *Know thyself*! – which has been passed down to us as philosophy's original motto. How, Socrates asks, *can *we know ourselves? In Socrates's own words, "how can we come to know the self itself [*tropon eurethein auto tauto*]" (129b)? It is a question of *how*, rather than a question of *what *– a question of form over content, and this is already a shift in rhetorical registers. The self or soul is not a "what," it is not some thing among things, it is not some knowable content subject to technology. Instead, as we shall learn, the self or soul is itself the *form *that makes knowledge possible. The fascinating question here is: in what way – *how *– does the self know itself? *How *does it reflect upon itself? *How *does the self relate to itself? What are the forms of such a relation? Or what are the *terms *by which the self will relate to itself? By "terms" here, I speak metaphorically: the self-self relation is mediated by terms that include words, images, concepts, values, etc. that are in circulation in the culture. This shift in approach from *what *to *how *– from *content *to *form *– is tremendously difficult for us to conceive because we moderns are trained to think propositionally, according to a disinterested epistemology that weighs the truth or falsity of some particular proposition or other. But Socrates poses a more vital question.

Socrates tells Alcibiades that in order to know oneself, one must *attend to the self*, in other words, one must take *care of the self – epimeleia heautou*. Again, for the Greeks, knowledge here must not be understood in the modern sense as propositional or as referring to mental evidence. Recall that for the Greeks there is not yet a sense of the "self" as we understand it; the self is not yet individuated, not yet a Cartesian, rational subject, not yet a source or origin of subjectivity. Instead, this ancient "self" is vital and relational, a political self whose self-relations and knowledge involve others originally: a self that is originally a relation. Knowing oneself, according to Socrates, is a relation of care, it is a *spiritual *practise, the form by which the self reflects upon itself. In Foucault's discussion of the *Alcibiades*, he emphasizes that care is a practise: " [it is] both exercise and meditation.... a number of actions exercised on the self by the self, actions by which one takes responsibility for oneself and by which one changes, purifies, transforms, and transfigures oneself" [25]. This means that the self is not in the first instance an epistemological subject; rather, the self is first and foremost a spiritual relation that will be the basis of any possible epistemological truth. One is what one does. In other words, the caring self-self relation is here the condition of possibility for any epistemic knowledge whatsoever. The self must first prepare itself spiritually for the reception of truth. This belief was passed down and has informed Christian ascetic practises, for example. Foucault formulates the self-self relation as a question: the self must *ask *itself, "How must I transform my own self so as to be able to have access to the truth" [25]? So to repeat, the spiritual relation the self has to itself will inform epistemological truths and falsities, not the other way around – epistemology is not the foundation of the self, as it has been since Descartes. This turns modern Western philosophy and politics upside down.

To elaborate this relation of care, we must pay careful attention to the text of Plato's *Alcibiades*. Here, the word for "care" is *epimeleia*, and the Liddell-Scott Ancient Greek Dictionary provides us with two definitions: (1) care for a thing or attention paid to a thing: one popular translation is "to take pains over" something; and (2) care understood as a public charge or commission. So we can see that care is a relation that is directed both within and without. It is an ethical relation because it has everything to do with one's ethos, with the way one lives one's life and conducts oneself with respect to oneself, to others, and to the world in general. It is about the good *life*, not the good *self*. And it is worth noting that later in the Greek tradition, *epimeleia *is often substituted with the word *therapeuein*, which is the root of our own word "therapy." So, we are right to read a therapeutic relation here, or even a nascent bioethics. It is the kind of relation typical of a living being who is always in flux, temporally, and whose relations can always be improved, and whose knowledge can always be expanded.

In the *Alcibiades*, Socrates says that *epimeleia *– care – is the art (*technē*) of "making better [*veltion poiē*]" (128b). It is not a "technology" in the modern sense; that is, it is not a technical tool to be used by the subject, but is part of that subject's very comportment in the world. The relation is a relation of *poiesis*, a relation of invention or creation. Care of the self is therefore the kind of relation one has to oneself when one takes pains, when one strives, when one works to make oneself better, creatively. There is a strange temporal ambivalence in the self-self relation: it has everything to do with the way Alcibiades comports himself and how he *will *comport himself. He must strive to become wise, to become other than he is right now; the self must recognize itself as somehow *un*wise in this moment and as empowered to *become wise*. Socrates is absolutely explicit: the skills that we use when caring for *particular *things, that is, for our possessions – and even our bodies, as the physician does – is not the same as the art that we would use when caring for the self itself (128d). This is why he says that the physician, in so far as he is a physician, does not "know" himself. The physician does not care for himself in this way. Socrates is suggesting that care and knowledge go hand in hand here because in order to care for something, and in order to "make it better" through *poiesis*, we must first have intimate knowledge of the "thing" in question (128e). Socrates says: "Could we ever know what art makes the man himself better [*techne veltio poiei auton*], if we were ignorant of what we ourselves are" (128e)?

With this narrative, I am suggesting a different model for selfhood than the self of modern medicine that knows itself biomedically, technologically, instrumentally. This different model for selfhood is not the knowing self of "self-care." So what "self" does the Delphic motto invoke when it says: "Know thyself!"? And how is this knowing-relation actually a caring-relation? In a fascinating passage, Socrates employs a visual/anatomical metaphor: "suppose that," he says, "instead of speaking to a *man*, [the Delphic inscription] said to the *eye *of one of us... 'See thyself!' – how would we understand such advice" (132d)? Poor Alcibiades is perplexed, but Socrates explains: "an eye will see itself if it observes another eye and looks at the best part of it, the part with which it can see" (133a). In other words, the eye will not just see a reflection of itself, nor will it simply see another eye it will take as analogous to itself, but in the intertwining relation, the eye will *see itself seeing*. The relation is more than simply reflective. Alcibiades suggests using a mirror, but Socrates will have none of it. The relation is chiasmatic, as the French phenomenologist Maurice Merleau-Ponty would say. It is a chiasmus because we cannot say – or see – with certainty where the eye's *seeing *passes over into its *being seen*: sight must become insight, and must comprehend both aspects of seeing-seen. It is worth citing Merleau-Ponty at length, here:

As soon as I see, it is necessary that the vision (as is so well indicated by the double meaning of the word) be doubled with a complementary vision or with another vision: myself seen from without, such as another would see me, installed in the midst of the visible, occupied in considering it from a certain spot.... [H]e who sees cannot possess the visible unless he is possessed by it, unless he *is of it*. [26]

This is to say that sight is never in simple possession of its object, but is possessed by it. Vision has a double sense. There is a reciprocity or reversibility that cannot be comprehended by instrumental or technical reason alone. Merleau-Ponty states that there is "a reversibility of the seeing and the visible.... a reversibility [that is] always imminent and never realized in fact" [26]. In other words, there is never a factic coincidence, never a fusion between the seeing and the visible or seen. There are, as it were, two "selves," which mark the ambivalence of the human subject. There is no abstract seer here, but to see is to be part of the visible itself: it is an immanent critique. Most famously, Merleau-Ponty uses the sense of touch to explain this phenomenon. When the left hand that touches the right hand – traditionally, "subject" touching "object" – it is impossible to say precisely when the left hand ceases touching and the right hand ceases being-touched; it is impossible to say when the relation reverses, and the left hand starts being-touched and the right hand starts to do the touching: "this reflection of the body upon itself always miscarries at the last moment" [26]. As I suggested, it is as if there are two "selves," and provocatively, Merleau-Ponty proposes the image of the body's two lips here (*ses deux lèvres*), full of erotic and verbal potential (erroneously translated as "two laps") (English 136/French 179).

With the "care of the self," I think we are justified in seeing the self's relation with itself as similarly chiasmatic. Care is not instrumental, it does not go in one direction, but is already implicated in its return-to-self. The caring self never quite coincides with itself: if it did, it would be a machine, perhaps, and ethical questions might never arise for it. The self coinciding with itself is the model of "self-care," where there is a technical "care" that amounts to uncritically following orders and "best practices." In contradistinction, Socrates's metaphor of the eye and its relation to sight can easily be understood as a relation of care, the "care of the self." A self will know itself only if it knows another self, and knows the best part of that self, the part by which it knows, the part that is wise. And such a knowledge can only be comprehended through care or love. Socrates's caring love is exemplary in this regard because he loves Alcibiades for *himself*, Socrates's care is not instrumentalizable, not technical. Indeed, we must say that Socrates cares for Alcibiades because he cares for Alcibiades's care of himself – it is a relation of a relation. As Socrates remarks: "Your lover is rather he who loves your soul" (131c). Remember, Alcibiades is now twenty, and all his lovers have fled because he is past his prime, unable to serve instrumentally as a sexual or erotic object of satisfaction.

My point in recounting the story of Alcibiades is to show how the relation of the self to itself – *auto to auto *– cannot be reduced to a *technē*, the way it is in the modern model of self-care. Instead, I am suggesting that the "care of the self" is the difficult promise of a new kind of ethics. We have to be careful not to call this a "foundation." The self relates to itself non-foundationally, non-substantially, and in this respect, we might be justified to invoke Socrates when he speaks of the "soul." The soul or "*psyche*" is dynamic and without substance; it is neither body nor mind, as these terms are traditionally understood; it is neither cognitive nor conceptual. Instead, we might call it a rhetorical device for plotting the relation between the self and itself, which includes the relation between the self and the other whose love and wisdom helps to bring that self into a caring proximity with itself.

## Parental care: ethical and grotesque reproduction

"...the hysterization of women, which involved a thorough medicalization of their bodies and sex, was carried out in the name of the responsibility they owed to the health of their children, the solidity of the family institution, and the safeguarding of society" [27].

Socrates's care is nurturing, much as we might hope to find in the parental relation of love and care. In the space remaining, I would like to turn now to the case of parental care in order to offer a concrete instance of the two modes of care that I have sketched and the two modes of selfhood that are associated with them. I have argued for a shift in subjectivity as a consequence of new medical technologies. Certainly, this is not new. Foucault famously argues that at the end of the nineteenth century we began to understand the self as somehow both medically and morally responsible not just for itself, but for its offspring. Through medical technologies and burgeoning medical and psychiatric knowledges of the day, the health of the individual started to become a "public health" concern, subject to the powers of an expanding medico-welfare complex. "Whence the medical – but also political – project of organizing a state management of marriages, births, and life expectancies; sex and its fertility had to be administered" [27]. Eugenics movements began to take hold, and citizens became informed by a discourse on perversion that helped to underpin eugenic concerns: individuals believed that "perversion" would result "in the depletion of one's line of descent – rickets in the children, the sterility of future generations" [27]. Unsurprisingly, women's bodies were most frequently targeted, hystericized, and medicalized; women were constituted foremost as bodily selves, a self reduced to a womb, a self that is responsible foremost to and for the future generations she would bear as extensions of herself.

If these subjective effects have not changed since the late nineteenth century, our biotechnological capacity to affect ourselves and future generations most certainly has. Foucault's argument must be updated with the genetic vocabularies and the new reproductive technologies that inform the self today. As Nikolas Rose has remarked:

Selfhood has become intrinsically somatic.... The new genomic and molecular vocabularies of ourselves – like earlier biomedical languages of intelligence, or depression, or "hormones" – are being incorporated within these relations of the somatic self to itself. [5]

The hysterization of the body has today been replaced by the geneticization of the body. Indeed, although we remain preoccupied by the spectre of "degenerescence" [27], and although we experience a "biological responsibility" [27] as citizens did at the turn of the last century, at the turn of the twenty-first century the medico-political project is expressed in genetic terms. The self understands itself and its moral agency in terms of a genetic self, a self whose bedrock of truth lies in its genes, its DNA. We are responsible to promote the "good gene" (however this is conceived in the current discourse) and we are irresponsible if we risk passing on the "bad gene." In some cases, we are constituted as irresponsible if we do not do everything in our power to eradicate the "bad gene," which includes availing oneself of every possible technology to ensure the health of the baby, of the next generation, and ultimately, of the species [28].

As I mentioned above, critics have come to see genetic medicine as part of a neo-liberal form of self-governance, where the self is encouraged to think of itself as freely relating to itself, much as an entrepreneur relates to her enterprise. This is the self of self-care. The self here becomes its own product, as it were – all the in the name of "freedom," of course – and yet that self is nevertheless increasingly beholden to a cadre of experts who provide the terms by which that self will, consequently, "freely" examine itself, work on itself, improve itself, and so on [5,19,29-33]. If this is not sinister enough, I have a very deep concern that, in the age of genomic medicine, this neo-liberal model has also come to infect our conception of parenthood. The issue is more complex than current fears surrounding the genetic engineering of "designer babies." Understandably, some might say, parents wish to give their children opportunities that they themselves never had: parents wish to provide the best environment or culture, the best schools, the greatest number of opportunities for their children to succeed in life. And from a genetic perspective, parents might "naturally" wish to pass on "the best" of themselves to their offspring, preserving what are culturally coded as "good genes" and eliminating the "bad genes." Preimplantation genetic diagnosis (PGD) is one reproductive technology that is making this a reality.

I consider these technologies – this technological relationship to one's children – as a form of "self-care," a self-managing extension of the self that borders on the grotesque. This is because the care of self-care is based on utility, it is a utilitarian ethic, a medicalized self-relation that is not freely defined by the self, but that is promoted and constrained by the norms of the medical, insurance, and pharmaceutical industries, by HMOs, and by models of profit-sharing. Here, despite the outward appearance of authentic and ethical care, we find that care is being redefined – care comes to be normalized, disciplined, technologized. The self, while it appears to be relating ethically, instead relies on the expert terms and "best practices" that are defined by the industry.

Although I am wary of biotechnology and of an increasingly state-administered biotech apparatus, I would like to distinguish my position from bioconservative critics whose aversion to biotechnology tends to be based on "fundamental" – or often fundamentalist – truths. One famous example of this position is Leon Kass, former Chairman of President George W. Bush's Council on Bioethics. He writes:

Thanks to technology a woman could declare herself free from the teleological meaning of her sexuality – as free as a man appears to be from his. Her menstrual cycle, since puberty a regular reminder of her natural maternal destiny, is now anovulatory and directed instead by her will and her medications, serving goals only of pleasure and convenience.... [S]he has, wittingly or not, begun to redefine the meaning of her own womanliness. [34]

One problem with such a position, as I see it, is its claim to express a foundational truth about human nature and kinship, about what a woman is, and about her biological destiny. Kass promotes fixed and unyielding terms in the self's understanding of itself and in its social relations more generally. Such social and political conservatism is especially pernicious when its agenda is peddled under the sovereign imprimatur of "Science." How are we meant to understand terms like "maternal instinct" or "maternal destiny"? Motherhood is not some biological or genetic response to the stimulus of the small infant. My own position is, I hope, more nuanced. I am wary of biotechnologies for reasons that stand opposed to most bioconservative critics. While bioconservatives see biotechnology as leading to a threatening proliferation of viable social subjectivities, and while they militate against such a proliferation, arguing for the value of traditional kinship positions, among other things, I myself contend that such a proliferation is essential to ethical life. I believe we ought to promote many terms and values to be tested and discussed. And so, on the face of it, my position would perhaps commit me to endorsing biotechnologies on the grounds that a proliferation of choices in the genetic marketplace will actually increase the viable terms by which a subject might live the good life. But this is not always the case. Indeed, I fear that the promise of biotechnology carries with it a false promise of diversity. In practice, I fear that increased choice in, say, the genetic marketplace may prove detrimental to truly progressive social and political projects. Ultimately, a proliferation of choices in the genetic marketplace will *not *unequivocally result in greater social and political diversity, but may instead result in more stringent norms, less diversity, and greater intolerance of all forms of difference, genetic and otherwise.

I believe that our neo-liberal culture of self-care, coupled with an increase in NRTs, has begun to foster a grotesque parental narcissism – a narcissism that was once merely thought of as social or cultural, but today is potentially genetic and irreversibly embodied. Within a neo-liberal frame that privileges the entrepreneurial self, I worry that parenthood will become just another project of parental *self*-improvement. It is tempting only to reproduce "the best" of oneself, to relate to oneself and to one's own genetic productivity as a relation of improvement. This vision is extreme, but the discourse that surrounds "good" and "bad" genes promotes rigid norms of acceptability – norms that define in advance what life is worthy of life, what life worthy of our love. In this model, I as a parent do not just hope for a child whom I will love; rather, I selfishly want a child I *can *love, a child who is worthy of my love. Within this rubric, we see our practical understanding of care shifting toward a narcissistic self-care that is invisibly governed by disciplinary norms, expert opinion, and the ethic of self-improvement.

This is to suggest that the baby functions as a cultural trope – literally, a turn, one mode by which the self turns upon itself or relates to itself. In his 1914 essay, "On Narcissism," Sigmund Freud argued that children were the means by which parents might fulfil narcissistic desires, effectively shaping their children in a particular image of themselves, a working and workable extension of themselves. According to Freud, the parents' narcissism results in "the compulsion to ascribe every perfection to the child – which sober reflection would find no occasion to do" [35]. The parent reproduces his or her own desires and locates them in the child:

the child shall have a better time than his parents; he shall not be subject to the necessities which they have recognized as paramount in life. Illness, death, renunciation of enjoyment, restrictions on his own will, shall not touch him; the laws of nature and of society shall be abrogated in his favour; he shall once more really be the centre and core of creation – "His Majesty the Baby", as we once fancied ourselves. The child shall fulfil those wishful dreams of the parents which they never carried out.... At the most touchy point in the narcissistic system, the immortality of the ego, which is so hard pressed by reality, security is achieved by taking refuge in the child. Parental love, which is so moving and at bottom so childish, is nothing but the parents' narcissism born again.... [35]

I offer Freud's words as a rhetorical provocation. To be sure, Freud's condemnation is polemical, and we might well question the "nothing but..." in the last sentence above. However, Freud does prompt us to think very seriously about the ways we have come to understand our children. Today we are acutely aware that we pass along our genes; perhaps we are less aware that our desires are bound up in them, but we would be wise to consider the ways that genetic discourses contribute to modern forms of narcissism. In the age of genomic medicine, Freud's depiction has lost none of its trenchancy. NRTs, in shaping our understanding of parenthood, serve to facilitate this narcissistic obsession with oneself, ostensibly in the name of another. How often do we hear the moral command to act "for the sake of the children"? And at the extreme, we will hear that the childless couple is "selfish," which belies the selfish narcissism of reproducing couples, as Freud understands it.

A more ethical stance might be to keep the baby as a trope – a figure which is mobile and always open to revision and redeployment, a kind of chiasmatic relation that is not first conceived according to parental terms. In this vein, we might succeed in further problematizing the parent-child relation, which is one effect of Freud's text. Bearing this text in mind, we might resist the appeal of a "genetic narcissism," the urge to understand our children as biological products whose bio-genetic makeup is now subject to our manipulation and desires. We must beware lest these manipulations and desires represent no more than a technologically advanced narcissism in the Freudian sense. And it is difficult to resist the implicit narcissism that NRTs foster. In the name of science, in the name of improvement, or even in the name of evolution, captured by Richard Dawkins's [36] controversial notion of "the selfish gene," we might be persuaded that our genes actually "want" us to make children in our own image, only better, brighter, healthier, faster.... We turn away from the wider implications of our choices: our responsibility to our children trumps our responsibility for the wider social and cultural effects that our choices may beget in some unforeseeable future. We get swept up in what some have called a "medical" or "reproductive fundamentalism" [37,38]. "Evolutionary" biology ought not to be the public face of – an apology for – what is essentially the preservation or reproduction of a particular way of life, a particular image of self, a particular social, economic, cultural, racial, or political identity. Of course, the language of the gene serves, conveniently, to mask these other realities.

To be clear, this is not a negative judgement on those parents who opt for genetic screening, for those who abort a foetus on genetic grounds, or those who use pre-implantation technologies in the effort to conceive a healthy child. Indeed, the burden of raising a congenitally sick or disabled child would be more than many people could reasonably bear – emotionally, socially, economically, and so on. However, as I suggested above, I do worry that with an increase in the availability and use of genetic screening and pre-implantation technologies, the definition of healthy and acceptable human life will become increasingly narrow. This is what Evelyn Fox Keller has described as a "eugenics of normalcy" [39], where socially unrealistic standards of normalcy put a new face on the old eugenics. Eliminating risk is now regarded as a (pre)parental duty, and while this project is promoted as fulfilling a "due care" that is presumably "owed" in advance to the foetus, it is not at all clear to me that this care is not in some sense a perversion of ethical love and care. Love, after all, involves risk. Love is a chiasmatic relation that is marked by uncertainty and by trust in the face of uncertainty. Love is a relation to a future that cannot be fully owned, controlled, anticipated. It is a promise of self with no guarantee that I will be returned to myself in quite the same terms. Regarding our children, then, an ethical relation of care is a relation that fosters the child's own development, encouraging a future that opens onto the unforeseeable, a proliferation of new terms and new modes by which that emerging self will relate to itself in its own tentative ways.

There are unavoidable social and political dangers when we presume to define parenthood in exclusively biological or genetic terms. On this ground, for instance, some critics argue against same-sex marriage and parenthood because they believe a child's right to know and to be raised by his or her *biological *parents is a right that supersedes adult rights – the rights of would-be parents, straight or gay, fertile or infertile. This is Margaret Somerville's position [40], who recently sparked protest at Toronto's Ryerson University when she was awarded an honorary Ph.D. in June 2006. Somerville problematically argues "the case of the child," whom she imagines is born with the desire both to know his or her biological parents and to be raised by them. She claims this as the child's fundamental "right," based on "nature," biology, and even genetics. Instead, I would see these desires as socio-historical conventions that take on a troubling authority when they are wholly biologized. We might stop ourselves to ask how it is that these imagined children's needs should trump the rights of would-be parents. This position, when taken to the extreme, will demand that we sacrifice the health and well-being of the mother for the sake of the child, whose sovereignty is constructed as sacred and ultimate. Worse, reducing parenthood to the so-called needs of the child – again, needs that Somerville describes as biological and "natural" – actually abrogates *true *parental responsibility in the name of performing some ideological and perverse version of it.

I am advancing a different view of parenthood here, an ethical view in which the child is cared for regardless of its biological heritage or genetic makeup. Somerville's moral position is clear: in the name of the child, we have a duty to prevent a whole sub-class of humans she calls "genetic orphans," whether these "orphans" are raised by same-sex couples or have come into this world thanks to sperm or ova donation or even adoption. But I fear that Somerville misses the point, ethically. If it is true that children suffer from "genetic orphanage," she never asks why: she presumes it is biological rather than social, the effect of some biological deviance rather than a social effect, for which she, too, bears some degree of responsibility. Rather than fetishizing genetic "rights," perhaps we ought to step back and reassess what parenthood really means in the myriad forms that it takes in our culture. Genetic essentialism too narrowly defines – and too dangerously limits – the ethic of care that we might hope to find in a good parent. I do not discount the desire of the adopted child to "know" his or her biological parents; however, I am critical of the whole cultural machinery – Somerville included – that overdetermines biological identity.

I am arguing here for a model of parental care based on the Socratic and Foucaultian "care of the self." This is an etho-political relation that ought not to be reduced to biology and epistemic certitude. But it is important to take pains to define how such a care will be realized. Several critics have warned against the potential political abuses of "care," suggesting that care "tends to be associated with conventional understandings of the family and biological connection" [41]. This is one way to impose a social and political agenda; it is a technological model of care in which "care" is never problematized, in part because a liberal political subject is presumed, a subject that understands care simply as an extension of his autonomy, according to paradigms of "self-care." Indeed, while the Canadian Royal Commission on NRTs was titled *Proceed with Care *[42], its debate over whether NRTs should be available to lesbians, for instance, belies the norms and constrains implicit in what "care" ought to be, and who, precisely, is deemed worthy or capable of caring in this way. Similarly, many political theorists argue for an ethic of care but nevertheless arrive at an impasse because they see care as a technology, that is, as a social and institutional practice [43,44]. I take issue with the sharp distinction between public and private in these arguments, however. These critics see care as normative or prescriptive – principles to be followed. For instance, Sevenhuijsen claims that "all definitions of care contain normative dimensions" [43]. But by the "care of the self," Foucault helps us to depart from this normativity. For him, care is a way of being-in-the-world, an attitude, a chiasmatic relation that constitutes the individual and the institution as two separate poles whose positions rely on dynamic power relations and norms that ought to be critiqued. Sevenhuijsen and Tronto erroneously start by presuming the givenness of individual selves and institutions responsible for our care; this is the model of "self-care" as I have defined it. In contradistinction, Foucault does not presume such a givenness. Thus, rather than seeking to meliorate these two poles – self and institution – we must think in terms of the implicit power-relations that define them and constitute them as "origins" in themselves. In a way, the relation is more significant, and the poles, derivative. The ethical self does not exist as a pre-constituted and self-contained, autonomous entity who would then use care as a technology; instead, she is constituted in and by her relations of care, where the relations inform the terms of that self.

One example for a more progressive model of care – structured on what I would call the "care of the self" – is discussed by Sara Ruddick. Much like Foucault, Ruddick understands care as open-ended, allowing for a proliferation of terms by which the self will relate to itself and to others. If we care for our children ethically, Ruddick suggests, then we will foster "conditions of respect for unpredictable and as yet unimagined difference and variety among and within people" [45]. This is an elaboration of the social psychologist Carol Gilligan's [46] work. Interestingly, Ruddick ultimately argues that such a model of care has applicability for an international politics of peace.

## Conclusion: care and questioning the good life

Emerging biotechnologies are sure to have social, political, and material effects the likes of which we have not yet even begun to imagine. I have argued that one effect is the death of traditional subjectivity, that is, the end of the principle of autonomy, the end of the rational, post-Enlightenment subject. Thanks in part to biotechnologies that promise to shape our elementary particles (to invoke the novelist Michel Houellebecq [47]), I believe that there can be no wholesale return to the foundational discourse of liberal humanism. While there may be good reasons to finally abandon our commitment to the sovereign subject, including its neoliberal instantiations, the challenges of biotechnology seem to make it inevitable. Whether we choose to mourn or to celebrate (each according to his politics), we cannot deny that this death leaves us quite without an adequate lexicon to understand political agency and without a traditional foundation for (biomedical) ethics. For this reason, analytic philosophers and public policy wonks alike have worked to reconstitute this traditional subject. But despite their efforts, through such movements as "self-care," increasingly we will find ourselves without a discourse that is commensurable with the new modes of subjectivity that are arising in tandem with burgeoning biotechnologies. This calls for a thoroughgoing reappraisal of subjective life and the etho-politics of the subject.

The "care of the self," as I have been sketching it, might be one way to begin to move beyond such an aporia, toward a different discourse on subjectivity – one that does not anxiously attempt to reinstate a sovereign, autonomous, and rational agent. To be clear, "care of the self" should be seen as a social and political project that does not condemn new genomic technologies out of hand; instead, it would be a critical project that returns us to the question of the self and the question of care in the pursuit of the good life. In other words, it would vitalize the questioning relation that the self has with itself, and it will look beyond, to question the kinds of subjects that emergent biotechnologies will inaugurate. It would refuse the absolute identity and oneness of the questioning self. It would keep this self-self relation open and dynamic – chiasmatic – rather than closing it epistemically or morally through reductionist genetic terminologies or other biotechnical fundamentalisms. In effect, I am arguing that being-human necessitates an ethical openness that biotechnologies – in many of their current discursive formulations at least – threaten to close rhetorically. Thus, I tend to agree with Heidegger and others who have argued that the real threat of such technologies lies not in the physical destruction of humanity, but in what we might call its spiritual or rhetorical dimension, an openness that is too easily closed when we intervene in our elementary particles to manipulate human physical and psychic features. Thus, the real horror is not that something will go wrong with these technologies, but that they will work too well. To cite Žižek, the danger is, "precisely, that ***nothing ****will go wrong*, that genetic manipulations will function smoothly – at this point, the circle will in a way be closed and the specific openness that characterizes being-human will be abolished" [10].

This is not to deny that biotechnologies could, in fact, foster a rhetorical and ethical openness. To take the example of NRTs, we might look to the ways that traditional kinship norms have been challenged and new conceptions of the family have emerged as a result. While these effects are promising, I believe they are rare; more frequently, we find a kind of operational bad faith in which biotechnologies are used instrumentally, and end up underwriting the fiction of a sovereign, rational, and autonomous subject. This is disastrous because such a fiction – surely hubristic – will blind us to the wider effects of our actions. Better, then, to proceed with an avowed unknowingness, rather than with the certainty of epistemic and moral closure that so often accompanies Science.

In brief, I am calling for a renewed discourse on the meaning of ethics and politics in the genomic age. What class of genes, what race, and what sexual orientation will be reproduced? And according to what – and whose – ideology of "the fittest"? Clearly, that which is reproduced through biotechnology is never merely biological: will we reproduce a horrific ideology or will we nurture an ethic of care? How ought we to relate to ourselves and to each other? How shall I understand my children, if the "truth" of that child is, first and foremost, genetic? Will I seek to pass on only my "best genes"? Would any less be less lovable? And why do I reproduce in the first place? Do I hope for a child I will love, or do I want a child I *can *love, a child worthy of my love, an image of myself, only better? What are the risks, and isn't risk, too, part of our humanness? When I love, am I not taking a risk, embarking on a relation that is open and inventive, unforeseeable and spontaneous, where I promise and there is no promise I will be given back to myself in quite the same way? Care, like love, ought to promote diversity. Rhetorically, it is a polysemic relation that, in an ethical moment, cedes control, and fosters inventive relations between the self and others, questioning normative constraints by allowing our creations to signify in their own ways. This is how we might define ethical parenthood: an ethic of trust, not to contain, but to allow and to love the new. Despite the temptation, promoted by traditional bioethics and public policy, we cannot seek recourse in a foundational autonomy, in the principles of abstract reason or utility, or in a self that uncritically does the bidding of those ideologies we call family, nation, or race – however "naturalized" or "biologized" these terms may become. An ethical care will mobilize these as the tropes that they are, and seek new relations, new modes, and new terms by which we might once again ask the question of the good life. This is a call for a renewed discourse that dwells not in identities but in relations, not in DNA but on the ways it is socially and politically and ethically constituted as meaningful.

I believe that we have arrived at a propitious moment in human history – propitious and necessary – in which to reopen the question of the self. Our technologies threaten to outstrip our capacity to develop both an adequate vocabulary and a liveable aesthetics of existence. What – or who – is a self? And what ought it to be? What, exactly, do we seek to reproduce in our use of NRTs? These questions engage the norms within which selfhood is circumscribed, the norms by which the self both governs itself and is governed by others. With genetic manipulation, we must ask whether the self becomes reified, essentialized, thing-like, a product. After all, there is something haphazard and open-ended in the way the self relates to itself, chiasmatically: this self-relation involves others, it is inventive, it is a relation of difference, and it is therefore political and historizing. Our self-relation is not an endless, and hence ahistorical, clone-like repetition of the same. Ethical freedom requires a spontaneity-of-self. Is the genetically manipulated child stripped of this freedom, rendered ghostly, lifeless? Freedom requires contingency and a certain unknowingness, a risk, even a struggle; human freedom is never fixed, always tentative; human life is life because death is at the frontier, a historizing event. The "care of the self" is, then, a response, but not a response that must choose between the false binaries acceptance/resistance, liberation/oppression, or nature/*technē*. Instead, the "care of the self" inaugurates a self that strives to open up a plurality of relations, a multiplicity of possibilities within which that self might relate caringly not only to itself, but to those others in its care. To care, in this respect, is to care for the care of others, to care for the modes by which they might, one day, come to care themselves, and not just in caring *for *themselves. This is not just a model of ethical parenthood, but an argument for ethical political relations, for the good life.
